# Engineered N-TIMP2 Variant Specifically Targeting MMP-9 Exhibits Potent Anti-Glioblastoma Activity

**DOI:** 10.3390/biom15101470

**Published:** 2025-10-17

**Authors:** Mark Feldman, Naama Rotenberg, Julia M. Shifman

**Affiliations:** Department of Biological Chemistry, The Alexander Silberman Institute of Life Sciences, The Hebrew University of Jerusalem, Jerusalem 9190401, Israel; mark.feldman@mail.huji.ac.il (M.F.);

**Keywords:** glioblastoma, matrix metalloproteinase 9 (MMP-9), tissue inhibitor of matrix metalloproteinase 2 (TIMP-2), MMP inhibitors, protein engineering

## Abstract

Glioblastoma (GB) is the most aggressive form of brain cancer. However, despite intensive intervention, GB almost invariably recurs due to the highly invasive nature of its tumor cells, which infiltrate surrounding healthy brain tissue, underscoring the urgent need for more effective therapies. One such approach could be based on targeting matrix metalloproteinase-9 (MMP-9), an enzyme that plays a crucial role in GB progression and is closely associated with enhanced invasiveness and poor prognosis. Previously, we engineered a potent and selective MMP-9 inhibitor derived from the N-terminal domain of the endogenous tissue inhibitor of metalloproteinases-2 (N-TIMP2). In this study, we evaluate the efficacy and toxicity of this engineered N-TIMP2 variant (REY) in adult GB U251 and normal Vero cells using multiple in vitro assays. Our results demonstrate that REY significantly inhibits colony formation and cell invasion, and markedly reduces spheroid spreading at nanomolar concentrations. Importantly, the engineered variant, which is highly specific for MMP-9, consistently outperforms the wild-type N-TIMP2, which broadly targets multiple MMPs, and exhibits no cytotoxicity toward healthy cells. Together, these findings support MMP-9 as a viable therapeutic target in GB and highlight the potential of our engineered N-TIMP2 variant as a promising candidate for further therapeutic development.

## 1. Introduction

Glioblastoma (GB) is the most common and lethal form of primary brain cancer that originates in glial cells and is characterized by an aggressive, fast-growing nature and a tendency to infiltrate surrounding healthy brain tissue. Despite treatment involving surgery, radiation, and chemotherapy, the average survival time of GB patients is 12–15 months after diagnosis [1]. A major challenge in GB treatment is the ability of the aggressive tumor cells to invade surrounding healthy tissues, resist apoptosis, promote angiogenesis, and escape the immune response [2]. Cancer cell spread and invasion are complex processes closely linked to the activity of matrix metalloproteinases (MMPs), a family of 23 human enzymes responsible for degrading the extracellular matrix. Several MMPs within this extensive family have been specifically implicated as key drivers of cancer malignancy and metastasis, making them promising targets for therapeutic intervention [3,4,5]. In particular, MMP-9 is a demonstrated driver of tumor progression in several types of cancer, including colorectal cancer, non-small cell lung cancer, triple-negative breast cancer, and others [6,7,8,9,10,11,12,13,14,15].

Recently, MMP-9 has been implicated in GB progression, recurrence, and treatment resistance [16,17,18,19]. MMP-9 expression is upregulated in glioma tissues, where it correlates with tumor grade [16], and its levels are significantly elevated in the serum of GB patients, suggesting its potential as a diagnostic marker for disease progression [19]. Blocking of MMP-9 expression with RNAi led to decreased invasiveness of glioma cells and increased response to chemotherapy [20,21]. Moreover, MMP-9 plays a major role in the disruption of the blood–brain barrier (BBB), significantly contributing to the tumor’s aggressive and invasive behavior [22,23,24,25]. Most importantly, recent research has demonstrated that both standard GB therapies, temozolomide (TMZ) and radiotherapy, stimulate MMP-9 overexpression and enhance cell invasiveness, further underscoring the role of MMP-9 in treatment resistance [17,18]. Thus, the mounting evidence points to MMP-9’s critical involvement in GB pathophysiology, making it a key focus in ongoing cancer research and therapeutic development.

Although numerous MMP inhibitors have been developed over the years [26,27,28,29], these compounds have been unsuccessful in clinical trials, primarily due to high toxicity resulting from cross-reactivity with other proteins or inefficacy resulting from binding to inactive forms of MMPs. Increasing evidence highlights the distinct roles that different MMPs play in various diseases, with some exhibiting protective effects [30,31,32,33]. This underscores the importance of designing selective inhibitors that target individual MMPs or a narrow subset, rather than broad-spectrum inhibition. An attractive strategy for specific MMP inhibition takes advantage of small endogenous proteins, tissue inhibitors of metalloproteinases (TIMPs). There are four TIMPs in the human genome that inhibit all MMP family members and function to prevent excessive extracellular matrix remodeling. TIMPs already possess high (pM to low nM) affinity to MMPs, no toxicity and immunogenicity, and could be converted into highly specific inhibitors of a particular MMP type. In the past decade, a number of studies reported TIMP variants with enhanced specificity for one of the MMPs [34,35,36,37,38]. Our group engineered a variant of the N-terminal domain of TIMP2 (N-TIMP2) called REY with eight mutations that inhibit the MMP-9 activity with ~100 pM *K_i_* while showing much weaker inhibition of other MMPs [39]. Furthermore, we demonstrated that REY exhibited superior inhibition of cell invasiveness and clonogenicity over WT N-TIMP2 in triple-negative breast cancer cells [39]. In this study, we examine the ability of REY to inhibit various pro-cancer processes in adult GB U251 cells using multiple in vitro assays and compare its inhibitory potency to that of WT N-TIMP2. We show that this protein effectively inhibits colony formation, invasiveness, and spread of tumor spheroids while exhibiting no cellular toxicity to healthy cells, thus making it an attractive drug candidate in GB.

## 2. Materials and Methods

### 2.1. N-TIMP2 Expression and Purification

The sequence of the WT N-TIMP2 WT and the REY mutant was cloned into the pPICZαA vector purchased from Genscript Inc, and expressed in *Pichia pastoris* as previously described [39]. The two proteins were purified by Ni-affinity chromatography followed by size-exclusion chromatography to obtain pure monomeric species. Proteins were kept in aliquots at −80 °C until usage.

### 2.2. Cell Lines

For the purpose of this study, we used the U251 GB cell line derived from a malignant glioblastoma tumor of a 75-year-old male and healthy Vero cells derived from monkey kidney epithelial cells. Both cell lines were grown in Dulbecco’s modified Eagle’s medium (DMEM) supplemented with 10% fetal bovine serum (FBS) and antibiotics at 37 °C in a humidified atmosphere of 5% CO_2_.

### 2.3. Colony Formation Assay

The assay was performed similarly to that described in our previous paper [39]. Briefly, human glioblastoma U251 cells were grown in Dulbecco’s modified Eagle’s medium (DMEM) supplemented with 10% fetal bovine serum (FBS) and antibiotics at 37 °C in a humidified atmosphere of 5% CO_2_. The U251 cells were seeded in a 24-well microplate (10^4^ cells per well), treated with different concentrations of either WT N-TIMP2 or REY mutant, and cultured in DMEM supplemented with 10% FBS for 2 days under standard conditions (37 °C and 5% CO_2_). Untreated cells were set as the control. After 48 h, the growing medium was replaced, and WT N-TIMP2 or REY mutant were readded to the medium under the appropriate concentrations, and the culture was maintained for 3 more days. After 3 days of incubation, the resulting colonies were fixed with 100% methanol for 20 min at −20 °C, stained with 0.4% crystal violet for 30 min at RT, and finally washed with double-distilled water to remove the excessive stain. Each sample was imaged using a CKX53 Olympus (Tokyo, Japan) inverted microscope at a magnification of 100×, and the colonies of each sample were counted using ImageJ software (version 3.91; Java image processing program; Rasband, W.S., ImageJ, U. S. National Institutes of Health, Bethesda, MD, USA) (https://imagej.net/ij/index.html, accessed on 1 May 2025). The assay was conducted in triplicate.

### 2.4. Cell Invasion Assay

Cell invasion was assayed via a Corning Matrigel invasion chamber 24-well plate using a similar method as described previously [39]. Briefly, Matrigel (CLS356234 Corning(R) Matrigel(R) Basement membrane) was diluted in cold PBS at a 1:4 ratio. The upper compartment of the transwell chamber was coated with 100 µL Matrigel and incubated overnight at 37 °C until gelled. In total, 2 × 10^5^ of confluent U 251 cells were resuspended in serum-free DMEM in the absence or presence of N-TIMP2 WT/REY at different concentrations and then transferred into the upper chamber of each transwell. Untreated cells were set as the control. Lower chambers contained fresh DMEM supplemented with 20% FBS. After culturing for 24 h, the cells at the upper surface of the membrane were removed using a swab, while the cells that migrated to the lower membrane surface were fixed with 100% cold methanol for 30 min and stained with 0.4% crystal violet for 1 h. The cells were imaged using a CKX53 Olympus inverted light microscope at a magnification of 100×, and the colonies of each sample were counted using ImageJ software (https://imagej.net/ij/index.html). Each experiment was repeated at least two times.

### 2.5. Spheroid Invasion Assay

First, U251 spheroids were formed using the hanging drop culture method, allowing single cells to aggregate and fabricate spheroids in the form of droplets. Briefly, 40 μL drops (104 cells/drop) of cultured U251 cells were deposited onto the bottom of the Petri dish lid. Then, 10 mL of PBS was added to the bottom of the dish and acted as a hydration chamber. The lid was inverted onto the PBS-filled bottom chamber and incubated at 37 °C, 5% CO_2_, in a humidified atmosphere for 4 days. In order to induce spheroid formation, we used 0.2% methyl cellulose (MC) culture medium [40,41,42]. Matured, compact spheroids were transferred to 24-well plates (single spheroid per well) pre-coated with 150 µL Matrigel as described above. DMEM containing various concentrations of N-TIMP2 WT/REY was added to each well, and the plates were incubated at 37 °C and 5% CO_2_ in a humidified atmosphere. After 3 days of incubation, spheroids were re-treated with WT N-TIMP2 or REY mutant under appropriate concentrations, and the plates were further incubated for an additional 6 days. The spheroids’ morphology and invasion capacity were monitored daily using a CKX53 Olympus inverted light microscope at a magnification of 40×. The assay was conducted in triplicate. The area invaded by cells leaving the spheroid was calculated using Image J software (https://imagej.net/ij/index.html) as the difference between the total invasion area, including spheroid and invaded cells and the area of the spheroid core. The assay was conducted in triplicate.

### 2.6. MTT (3-(4,5-Dimethylthiazol-2-Vl)-2,5-Diphenyl Tetrazolium Bromide) Assay

The N-TIMP2 variants were tested for in vitro cytotoxicity, using U251 (Brain cancer) and Vero (Normal, African green monkey kidney; RRID:CVCL_0059) cells via a conventional MTT assay. Briefly, the above cell lines were incubated at a density of 5 × 10^4^ cells/well in 96-well plates for 24 h in 100 mL of DMEM with 10% FBS, at 37 °C in a humidified 5% CO_2_ incubator. Following seeding, the culture supernatant was removed, serum-free DMEM containing various concentrations of N-TIMP2 variants, REY and WT, was added, and the cells were incubated for an additional 24 h. Wells without N-TIMP2 variants served as controls. A total of 10 µL of MTT dye (5 mg/mL) was added to each well, and the plate was incubated for an additional 4 h. The medium, together with MTT, was aspirated off the wells, dimethylsulfoxide (100 µL) was added, and the plates were shaken for 5 min. The absorbance of each well was measured at 540 nm in a microplate reader, and the percentage of cell viability was calculated using the formula: Cell viability = average absorbance of six replicates containing N-TIMP2 variant/average absorbance of six replicates of control wells × 100%.

### 2.7. Enzymatic Inhibition Assay

The assay was performed similarly to that described previously [39,43]. Briefly, conditioned medium of U251 cells treated with different concentrations of N-TIMP2 variants was collected after 24 h and concentrated using Amicon^®^ Ultra Centrifugal Filter, 10 kDa MWCO (Merck, Rahway, NJ 07065 USA). Concentrated supernatants were mixed with MMP-9 fluorogenic substrate (Abcam, Cambridge, United Kingdom) in a buffer (50 mM Tris–HCl, 0.15 M NaCl, 10 mM CaCl2, and 0.02% Brij 35, pH 7.5) in black 96-well plates. Fluorescence at 395 nm was immediately measured at 37 °C, at least every 30 s for at least 1 h 30 min, with irradiation at 325 nm, on a Biotek Synergy H1 plate reader (BioTek, Winooski, USA). A sample containing growth medium diluted in buffer was used to measure basal fluorescence, and a sample containing supernatant without inhibitor was used to measure uninhibited enzyme activity. Broad-range MMP inhibitor prinomastat (Merck) was used as an additional control. Mean velocity (Vm) of enzymatic cleavage was calculated for each sample from the fluorescence data. The percentage of MMP-9 activity was calculated as Vm of each treated sample normalized to Vm of the untreated control.

## 3. Results

Using the previously developed protocols, we expressed and purified two N-TIMP2 variants, WT N-TIMP2 and the REY mutant [39]. Protein molecular weight was confirmed by mass spectroscopy, and its activity was verified by performing an MMP-9 enzyme activity assay with our protein acting as a competitive inhibitor [39]. We chose to test the activity of our N-TIMP2 variants in adult GB U251 cells, a cell line whose motility and invasion have been linked to MMP-9 activity and is widely utilized in GB research [44,45]. Notably, treatment of U251 cells with TMZ, the standard GB drug, has been reported to further increase the expression of active MMP-9 [18]. Healthy Vero cells were included in order to test the cytotoxicity of N-TIMP2 variants.

### 3.1. N-TIMP2 Variants Inhibit Cell Survival and Proliferation

**Figure 1:**
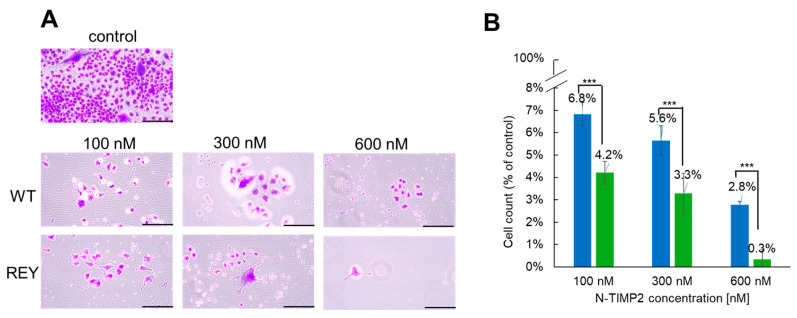
Inhibition of GB U251 cell clonogenicity by N-TIMP2 variants. (**A**) Images of U251 cell colonies treated with different concentrations of N-TIMP2 variants. Control represents untreated cells. The scale bar is 100 µM. (**B**) Images were analyzed with ImageJ software to count the colonies and to plot a bar graph. Colony count was normalized to that of the untreated control and plotted for WT N-TIMP2 (blue) and REY (green). Each experiment was repeated at least three times. *** indicates a *p* value of <0.05 in the standard *t*-test.

We first assessed the capability of WT N-TIMP2 and REY to inhibit cell survival and proliferation activity of U251 cells using the colony formation assay. The N-TIMP2 variant (either WT or REY) was added to the cells at different concentrations, ranging from 100 to 600 nM, as this range of concentrations has been shown to be effective in various assays on triple-negative breast cancer cells [39] (see Methods). Figure 1A,B show that both WT N-TIMP2 and REY exhibit strong inhibitory activity on colony formation of the U251 cells. All tested concentrations of WT and REY were able to decrease the number of colonies by more than 90% compared to the untreated control (Figure 1A,B). However, the REY mutant exhibited significantly stronger inhibitory effect at all concentrations tested compared to WT N-TIMP2. Finally, only 0.3% of colonies were formed compared to the untreated control after treatment of U251 cells with the highest tested concentration of REY (600 nM), indicating total suppression of clonogenicity.

### 3.2. N-TIMP2 Variants Inhibit Cell Invasion

**Figure 2:**
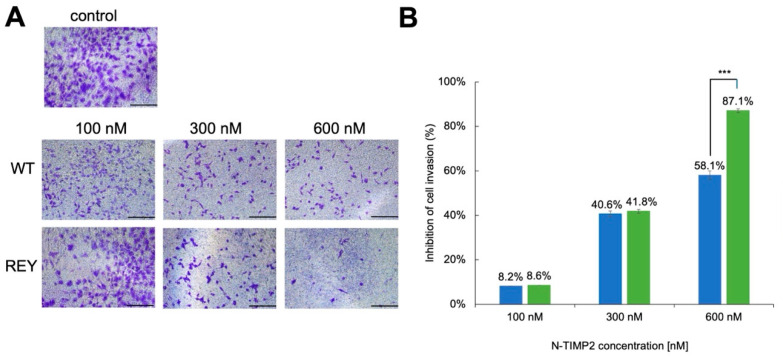
Inhibition of U251 cell invasion by N-TIMP2 variants. (**A**) Images of U251 cells invaded through Matrigel treated with different concentrations of N-TIMP2 variants. Control represents untreated cells. The scale bar is 100 μm. (**B**) The images in (**A**) were analyzed with ImageJ to count the cells and create a bar plot showing the percentage of U251 cell invasion upon exposure to different concentrations of REY (green) and WT N-TIMP2 (blue). The results were normalized to the control (untreated cells), which was set to 100% cell invasion. Each experiment was repeated 2–3 times. *** indicates a *p* value of <0.05.

Multiple studies implicated MMP-9 in the invasion and migration of tumor cells. To investigate the impact of MMP-9 inhibition on the invasion of U251 GB cells, we performed Matrigel transwell assays that measure the ability of cancer cells to migrate through an ECM barrier. These assays were performed in the presence of various concentrations of either WT N-TIMP2 or the REY variant and compared to a control that contained no inhibitor. Figure 2 shows that at the lowest tested concentration of 100 nM, both WT N-TIMP2 and REY exerted a minor inhibitory effect on brain cancer cell invasion, producing about 8% inhibition. When the concentration of the inhibitor was raised to 300 nM, the inhibition for both constructs was elevated to about 40%. Finally, at the highest tested concentration of 600 nM, both mutants exhibited substantial inhibition of cell invasion, with the REY mutant significantly outperforming the WT N-TIMP2 with 87.1% and 58.1% inhibition, respectively.

### 3.3. N-TIMP2 Variants Inhibit U251 Spheroid Spread

**Figure 3:**
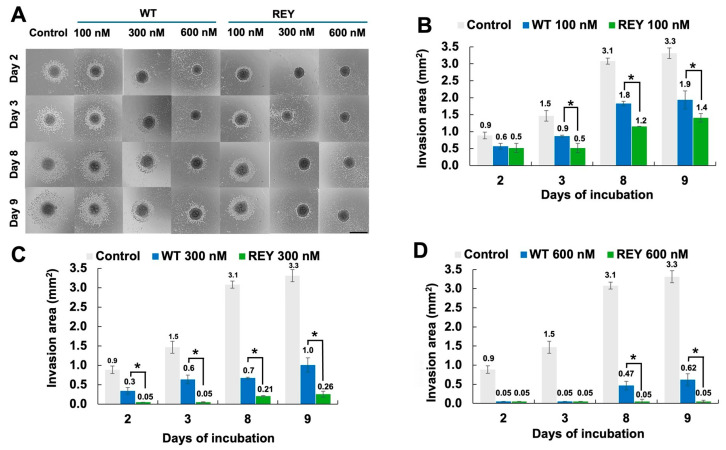
N-TIMP2 inhibition of spheroid invasion. (**A**). Images of spheroid invasion kinetics through Matrigel during 9 days of incubation. Spheroids were treated with different concentrations of N-TIMP2 variants. Day 0 indicates formed pre-treated spheroids. Control represents untreated spheroids. The scale bar is 1 mm. (**B**–**D**). The images were analyzed with Image J software to determine the invasion area of U251 spheroids upon treatment with different concentrations of REY (green) and WT N-TIMP2 (blue), compared to untreated control (gray) and between variants. The results were presented in absolute units as mm^2^. Each experiment was repeated at least three times. * indicates a *p* value of <0.05.

In the next experiment, we measured the activity of N-TIMP2 variants on the U251 spheroids, since spheroids better replicate the 3D structure and microenvironment of actual tumors compared to traditional 2D cell cultures [46]. For this experiment, we first formed U251 spheroids using the hanging drop method [47], subsequently transferred them to a 24-microwell plate containing Matrigel, and treated them with various concentrations of an N-TIMP2 variant at day 0 and repeatedly at day 3. The spread of cells from spheroids was subsequently analyzed over a 9-day period and compared across different protein variants and concentrations. As shown in Figure 3, both WT N-TIMP2 and the REY mutant significantly inhibited spheroid spread (i.e., invasion) compared to the untreated control, with the degree of inhibition increasing with concentration. Notably, U251 cell invasion was significantly more suppressed by the REY mutant compared to WT N-TIMP2, and this difference became more pronounced over time (Figure 3A–D). On day 9, for instance, spheroids treated with the lowest REY concentration (100 nM) exhibited an invasion area of 1.4 mm^2^, compared to 1.9 mm^2^ for those treated with WT N-TIMP2 (Figure 3B). At a medium concentration of 300 nM, the invasion areas were 0.3 mm^2^ for REY and 1.0 mm^2^ for WT N-TIMP2 (Figure 3C). Most strikingly, at the highest concentration tested of 600 nM, the REY variant almost completely inhibited spheroid invasion at all time points, while WT N-TIMP2 still permitted some spread, showing an invasion area of 0.6 mm^2^ on day 9 (Figure 3D).

### 3.4. N-TIMP2 Variants Are Not Cytotoxic to U251 GB Cells and Healthy Vero Cells

**Figure 4:**
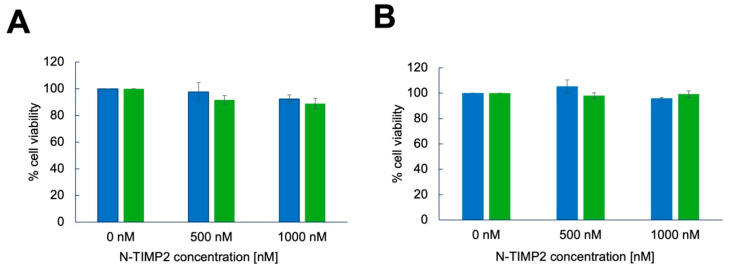
Effect of N-TIMP2 variants on cell viability. U251 GB cells (**A**) and Vero cells (**B**) were incubated with REY (green) or WT N-TIMP2 (blue) at the specified concentration for 24 h and the MTT assay was performed by adding the MTT substrate and measuring absorbance at 540 nm.

We next evaluated the effect of N-TIMP2 variants on the viability of GB U251 cells and non-cancerous Vero cells. For this experiment, we used an MTT assay that measures cell viability by assessing the reduction of the MTT reagent to formazan by metabolically active cells, with the resulting color change indicating the number of viable cells. Here, we added the N-TIMP2 variants at concentrations ranging from 0 to 1000 nM and measured cell viability after 24 h. Figure 4 shows that the viability of both GB U251 and Vero cells did not decrease upon incubation with either WT N-TIMP2 or REY, even at the highest tested concentration of 1000 nM. These findings demonstrate that N-TIMP2 variants do not cause cytotoxicity to GB U251 and non-cancerous Vero cells at the concentrations used in this study.

### 3.5. N-TIMP2 Variants Inhibit Enzymatic Activity of MMP-9 Released by U251 Cells

**Figure 5:**
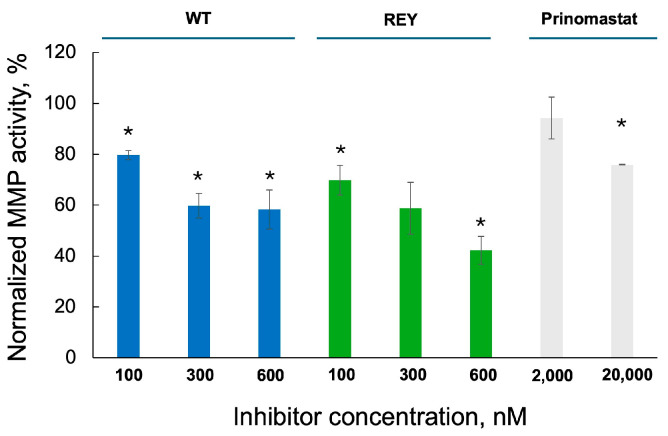
Inhibition of enzymatic activity of MMP-9 released by GB U251 cells by N-TIMP2 variants and prinomastat. Activity of MMP-9 in concentrated supernatants of U251 cells treated with N-TIMP2 variants and prinomastat was analyzed using an enzymatic activity assay. Data are presented as a percentage of MMP-9 activity treated by an inhibitor relative to its activity in the untreated sample. Each experiment was repeated at least two times. * indicates significantly lower activity relative to the untreated sample (*p* value of <0.05 in the standard *t*-test).

To prove that the anticancer effects we observed are due to inhibition of MMP-9, we measured the activity of MMP-9 released by U251 cells in the absence and in the presence of N-TIMP2 variants. For this purpose, we used an enzymatic activity assay with an MMP-9 peptide substrate that becomes fluorescent upon cleavage by MMP-9 (Figure 5). Our results demonstrate that U251 cells release active MMP-9. Furthermore, we observe dose-dependent inhibition of MMP-9 activity by both WT N-TIMP2 and REY constructs. MMP-9 activity in the samples treated with WT N-TIMP2 at 100 nM, 300 nM, and 600 nM was inhibited by 21%, 41%, and 41%, respectively, compared to the untreated control. The REY mutant exhibited an even more pronounced inhibitory effect as exposure of the samples to 100 nM, 300 nM, and 600 nM of REY produced inhibition by 30%, 41%, and 58%, respectively, compared to the untreated control. In comparison, the broad-range MMP inhibitor, prinomastat, demonstrated significant inhibition of released MMP-9 only at a much higher concentration of 20 µM. This lower potency of prinomastat could be due to its lower intrinsic MMP-9 affinity and to undesired interactions with other MMPs and homologous proteins, effectively reducing its concentration, thus proving that our REY variant is superior in inhibition of the U251-released MMP-9 compared to prinomastat.

## 4. Discussion

GB is a highly aggressive brain cancer that lacks effective therapeutic strategies for treatment. MMP-9 is significantly overexpressed in GB tumors and plays a pivotal role in disease progression by promoting tumor cell migration and invasion, enhancing angiogenesis, and disrupting the integrity of the blood–brain barrier (BBB). Notably, standard GB treatments such as radiation and temozolomide (TMZ) further upregulate MMP-9 expression, potentially exacerbating tumor aggressiveness. We thus postulated that specific inhibition of this enzyme would present an attractive strategy to counteract GM progression.

Our results demonstrate that inhibition of MMP-9 by the endogenous MMP inhibitor N-TIMP2 and its engineered variant REY effectively suppresses cancer cell proliferation, invasion, and spheroid spread in U251 GB cells. We further show that our N-TIMP2 variants inhibit the MMP-9 released by the U251 cells in a dose-dependent manner. These findings strongly support MMP-9 as a promising therapeutic target in GB, while also leaving open the possibility that additional interactions of N-TIMP2 variants contribute to their anticancer activity. Moreover, both WT N-TIMP2 and REY exhibited significant activity at relatively low concentrations (100–600 nM), with REY showing markedly greater potency across all assays. This enhanced efficacy is attributed to REY’s increased specificity and affinity for MMP-9, coupled with reduced binding to off-target MMPs, enabling more effective inhibition of MMP-9 compared to WT N-TIMP2 at equivalent concentrations.

While here we examined the effect of engineered N-TIMP2 variants on U251 cells, GB is characterized by high heterogeneity, thus pointing to the importance of investigating the effects of N-TIMP2 variants in different GB cell lines. Although we did not perform these experiments in this study, we anticipate that REY will exhibit similar anti-migratory and anti-invasive effects in other GB models. This hypothesis is supported by a recent study, where engineered minimal variants of other TIMP family members, TIMP1 and TIMP3, have been shown to reduce the migratory and invasive capabilities of additional GB cell lines, T98G and A172, which also overexpress MMP-9 [23]. These findings further support our hypothesis that MMP-9 is a critical therapeutic target in GB. Notably, the authors of that study reported no cytotoxic effects of their engineered TIMPs on GB cells at submicromolar concentrations—consistent with our observations. Similarly, we demonstrated that the REY variant exhibits no detectable toxicity in non-cancerous Vero cells at concentrations up to 1 μM, further underscoring the potential of engineered TIMPs as safe therapeutic candidates. Since Vero cells are of monkey origin, additional experiments assessing the toxicity of N-TIMP2 variants in healthy human glial cells, such as astrocytes, could provide stronger evidence for the safety of our drug candidate. We expect our variants to be non-toxic, as TIMP-2 is constitutively expressed by astrocytes [48], and recent studies have linked TIMP-2 treatment to beneficial neural effects in aging models [49].

In contrast, the standard chemotherapeutic agent TMZ displays high cytotoxicity toward both cancerous and non-cancerous cells [50]. Moreover, administration of TMZ results in adverse effects such as myelosuppression [51] and hematologic toxicity, including acute lymphoblastic leukemia [52,53]. The adverse effects of TMZ and fast-growing drug resistance motivate the search for more selective and less toxic treatments for GB.

## 5. Conclusions

In conclusion, this study presents strong evidence supporting the potential of our engineered N-TIMP2 variant as a novel therapeutic strategy for GB. This variant demonstrates low cytotoxicity and high selectivity for MMP-9, a previously uninvestigated GB target that is upregulated following standard GB therapy. In addition, its relatively small size facilitates cost-effective production and enhances tissue penetration compared to MMP-9-targeting antibodies. Future research should test the activity of this variant in additional GB cells and prioritize the long-term efficacy and safety of this protein in in vivo GB studies. Moreover, efforts should be directed toward developing advanced delivery systems to enable targeting of this protein to the brain utilizing the most advanced protein delivery methodologies such as nanoparticles, hydrogels and intranasal brain delivery. By directly addressing the highly invasive and treatment-resistant nature of GB, these innovative therapies could substantially improve patient outcomes and quality of life, representing a significant advancement in the fight against this aggressive brain cancer.

## Data Availability

Data generated in this study are available within the article or from the corresponding author upon reasonable request.

## Abbreviations

The following abbreviations are used in this manuscript:

GB	Glioblastoma
MMP	Matrix metalloproteinase
TMZ	Temozolomide
WT	Wild type
BBB	Blood–brain barrier
ECM	Extracellular matrix
N-TIMP2	N-terminal domain of tissue inhibitor of metalloproteinase
MTT	3-(4,5-dimethylthiazol-2-vl)-2,5-diphenyl tetrazolium bromide
